# Regulatory Effect of General Anesthetics on Activity of Potassium Channels

**DOI:** 10.1007/s12264-018-0239-1

**Published:** 2018-06-13

**Authors:** Yan Li, Jie Xu, Yun Xu, Xiao-Yun Zhao, Ye Liu, Jie Wang, Guang-Ming Wang, Yan-Tian Lv, Qiong-Yao Tang, Zhe Zhang

**Affiliations:** 1 Jiangsu Province Key Laboratory of Anesthesiology, Xuzhou Medical University, Xuzhou, 221004 China; 2 Jiangsu Province Key Laboratory of Anesthesia and Analgesia Application Technology, Xuzhou Medical University, Xuzhou, 221004 China

**Keywords:** General anesthesia, Potassium channel, Ion channel

## Abstract

General anesthesia is an unconscious state induced by anesthetics for surgery. The molecular targets and cellular mechanisms of general anesthetics in the mammalian nervous system have been investigated during past decades. In recent years, K^+^ channels have been identified as important targets of both volatile and intravenous anesthetics. This review covers achievements that have been made both on the regulatory effect of general anesthetics on the activity of K^+^ channels and their underlying mechanisms. Advances in research on the modulation of K^+^ channels by general anesthetics are summarized and categorized according to four large K^+^ channel families based on their amino-acid sequence homology. In addition, research achievements on the roles of K^+^ channels in general anesthesia *in vivo*, especially with regard to studies using mice with K^+^ channel knockout, are particularly emphasized.

## Introduction

Since the early 19th century, general anesthetics have been used to induce a state of unconsciousness for surgery. Modern anesthesiology defines a complete general anesthetic effect as including unconsciousness, amnesia, analgesia, and muscle relaxation that is indispensable for modern surgery. Hitherto, myriad molecular targets of general anesthetics have been identified, such as gamma-aminobutyric acid receptor, the N-methyl-D-aspartate receptor families, and ion channels [1–6]. Among the many kinds of ion channels, potassium (K^+^) channels are the most diverse and ubiquitous, playing important roles in controlling neuronal excitability and neurotransmitter release in the central nervous system by determining the membrane potential of neurons [7–12]. Thus, many studies have been conducted on the regulatory effects of general anesthetics on K^+^ channel activity. Also, data on these effects have accumulated, and this review summarizes recent research achievements in this area, especially emphasizing studies using K^+^ channel knockout (KO) mice. K^+^ channels that have been studied as general anesthetic targets have been assigned to 4 categories according to a standard nomenclature by The International Union of Basic and Clinical Pharmacology (IUPHAR) and the Human Genome Organisation Gene Nomenclature Committee (HGNC) [7]. They are voltage-gated (Kv) channels, the background/leak or tandem 2-pore (K2P) families, inwardly-rectifying (Kir) channels, and Ca^2+^-activated (K_Ca_) channels. Research advances related to the effects of general anesthetics on these channels are summarized based on the above classification.

## Modulation of Kv Channels by General Anesthetics

**Fig. 1:**
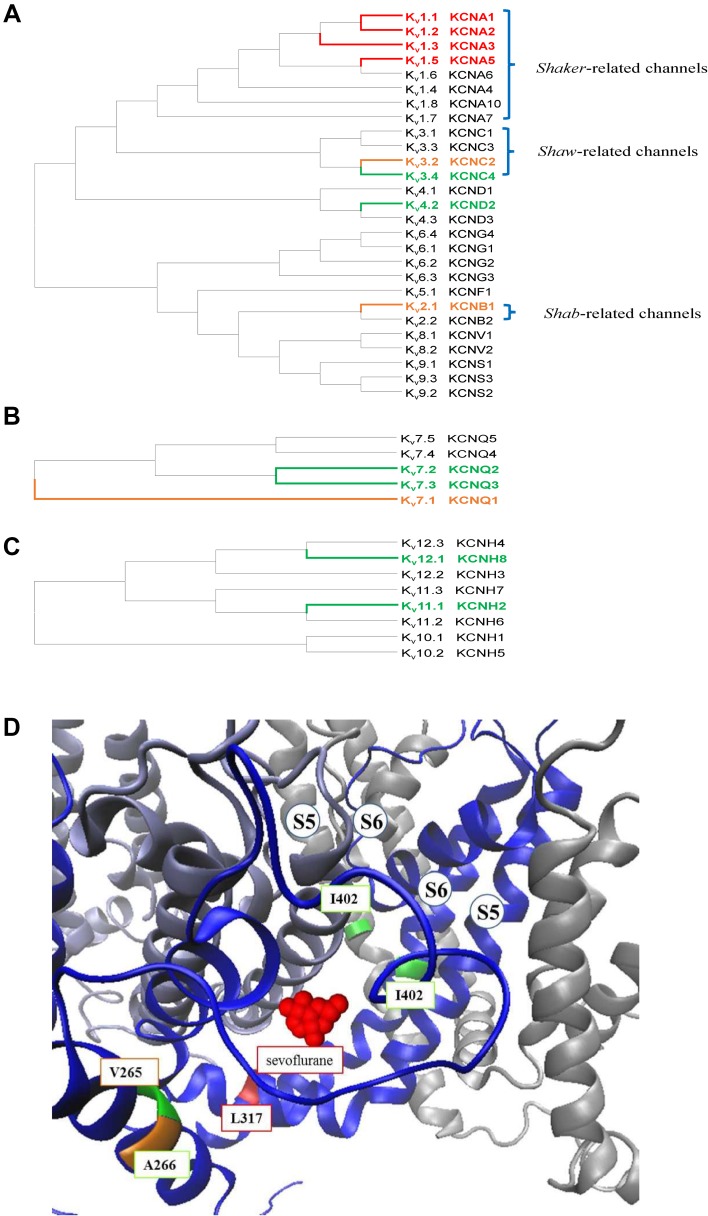
Phylogenetic trees of the Kv channel family and critical structure of Kv1.2 channel for sevoflurane binding. **A** Amino acid sequences of Kv1–9 channels were aligned by MEGA software with the Clustal W method and analyzed by the neighbor joining test. **B**, **C** Kv7 subfamily and Kv 10–12 subfamilies are shown separately because of the low amino acid sequence similarity with other Kv channels. Names of channel subtypes are labeled in color based on their different responses to volatile anesthetics in experiments performed *in vitro* with patch clamp. The channels that can be activated by volatile anesthetics are shown in red; channels inhibited by volatile anesthetics are shown in yellow; and channels insensitive to volatile anesthetics are shown in green. Channels with unknown responses to volatile anesthetics are in black color. The protein IDs of Kv channels used in this sequence alignment are as follows: K_v_1.1 (NP_000208), K_v_1.2 (NP_004965), K_v_1.3 (NP_002223), K_v_1.4 (NP_002224), K_v_1.5 (NP_002225), K_v_1.6 (NP_002226), K_v_1.7 (NP_114092), K_v_1.8 (NP_005540); K_v_2.1 (NP_004966), K_v_2.2 (NP_004761), K_v_3.1 (NP_004967), K_v_3.2 (NP_631875), K_v_3.3 (NP_004968), K_v_3.4 (NP_004969), K_v_4.1 (NP_004970), K_v_4.2 (NP_036413), K_v_4.3 (NP_004971); K_v_5.1 (NP_002227), K_v_6.1 (NP_002228), K_v_6.2 (NP_036415), K_v_6.3 (NP_579875), K_v_6.4 (NP_758857), K_v_7.1 (NP_000209), K_v_7.2 (NP_742105), K_v_7.3 (NP_004510), K_v_7.4 (NP_004691), K_v_7.5 (NP_062816), K_v_8.1 (NP_055194), K_v_8.2 (NP_598004); K_v_9.1 (NP_002242), K_v_9.2 (NP_065748), K_v_9.3 (NP_002243); K_v_10.1 (NP_758872) K_v_10.2 (NP_647479); K_v_11.1 (NP_000229), K_v_11.2 (NP_110416), K_v_11.3 (NP_150375); K_v_12.1 (NP_653234), K_v_12.2 (NP_036416), and K_v_12.3 (NP_036417). **D** The local structure of sevoflurane-binding site on Kv1.2 channels. I402 on S6 and the L317 in the S4–S5 linker form a sevoflurane (shown in red)-binding pocket with V265 and A266. Two domains with S5 and S6 linker are shown. The picture was drawn with the VMD software based on crystal structure of Kv1.2 with PDB file 3LUT.

The opening of Kv channels is regulated by a membrane potential change and triggered by moving of the voltage sensor domain located on the S4 segment. The voltage-gated Kv channel family includes 40 genes encoding pore-forming subunits that are divided into 12 subfamilies (Kv1–Kv12) based on sequence homology (Fig. 1A–C). The HGNC and IUPHAR nomenclature and names based on the homologous channels in *Drosophila* are shown in Fig. 1A [13].

### Members of the Shaker-Related K^+^ Channel Family (Kv1.1–Kv1.6) are Important Targets of Volatile Anesthetics

The Shaker channel was the first cloned voltage-dependent K^+^ channel from *Drosophila* [14, 15]. Flies with a mutated Shaker gene shake their legs under ether anesthesia (hence the name). Subsequently, Shaker-related K^+^ channels were cloned from mammals and named the Kv1.x channel family.

Given the critical role Kv channels play in limiting neuronal excitability, studies of the effects of general anesthetics on the Shaker channel began almost immediately after it was cloned. The earliest study performed on *Xenopus* oocytes revealed that chloroform and halothane reduce the macroscopic conductance of the Shaker channel while isoflurane increases its macroscopic conductance [16]. Consistent with these results, subsequent studies on *Drosophila* showed that halothane alters the electroretinogram by reducing its transient component at light-off *via* inactivation of the Shaker channel [17]. Meanwhile, the sleep state, which differs from anesthesia but shares some of its characteristics, is also shorter in *Drosophila* with a mutated Shaker channel [18]. These results suggested that Shaker-related K^+^ channels are important targets of volatile anesthetics (Table 1).

**Table 1:** The regulatory effects of volatile general anesthetics on potassium channels.

	Halothane	Isoflurane	Sevoflurane	Enflurane	Desflurane	Chloroform	Diethyl Ether	Ethanol	Xenon	Nitrous Oxide	Cyclopropane
K_v_1.1/1.2/1.3/1.5	Inhibition	Activation	Activation	–	–	Inhibition	–	–	No response	–	–
K_v_2.1	Inhibition	–	–	–	–	–	–	–	–	–	–
K_v_3.2	Inhibition	Inhibition	Activation	–	Inhibition	Inhibition	–	–	–	–	–
Kv3.4	–	–	No response	–	–	–	–	–	–	–	–
Kv4.2	–	–	No response	–	–	–	–	–	–	–	–
K_v_7.1	–	–	Inhibition	–	–	–	–	–	–	–	–
K_v_7.2/7.3	No response	No response	–	No response	–	–	–	Inhibition	–	No response	–
K_v_11.1	No response	No response	–	No response	–	–	–	Inhibition	–	No response	–
K_v_12.1	No response	No response	–	No response	–	–	–	No response	–	No response	–
TASK-1/3	Activation	Activation	–	–	–	Inhibition	Inhibition	–	No response	No response	No response
TREK-1/2	Activation	Activation	Activation	–	Activation	Activation	Activation	–	Activation	Activation	Activation
TRAAK	No response	No response	–	–	–	No response	No response	–	–	–	–
TASK-2	Activation	–		Activation	Activation	Activation	–	–	–	–	–
TALK1/2	Inhibition	Inhibition	–	–	–	Inhibition	–	–	–	–	–
THIK-1	Inhibition	–	–	–	–	–	–	–	–	–	–
TRESK	Activation	Activation	Activation	–	Activation	–	–	–	–	–	–
GIRK1/2 and GIRK2	Inhibition	Inhibition	–	Inhibition	–	–	–	Activation	–	Activation	–
GIRK1/4	Activate basal current but inhibit agonist-induced current	No response	–	No response	–	–	–	Activation	–	Activation	–
Kir1.1 and Kir2.1	No response	No response	–	No response	–	–	–	No response	–	No response	–
BK	Inhibition	Inhibition	–	Inhibition	–	–	–		–	–	–
IK	Inhibition	Inhibition	Inhibition	Inhibition	–	–	–		–	–	–
SK	No response	–	–	–	–	–	–		–	–	–

– unknown

Since then, the effects of general anesthetics on Shaker-related K^+^ channels in mammals have been widely studied. Barber *et al*. reported that 1 mmol/L sevoflurane potentiates *Drosophila* Shaker B, Kv1.2, and Kv1.5 channel currents over the physiological range of membrane potential (−60 to −40 mV) [19]. These results were further confirmed and expanded by a subsequent study by Lioudyno *et al*., who reported that a sub-surgical dose of sevoflurane (0.2 mmol/L) potentiates the currents of Kv1.1, Kv1.2, Kv1.3, and Kv1.5 channels at low depolarizing potentials (−40 to 0 mV) in a heterologous expression system. But at higher depolarization potentials (30 to 60 mV), the currents of Kv1.1 and Kv1.2 channels are still potentiated, whereas the Kv1.3 and Kv1.5 currents are inhibited [20]. The mechanism of action of sevoflurane on Kv channels can be explained as sevoflurane favoring the open state of Kv1.3 and Kv1.5 channels but accelerating its inactivation at high membrane potentials. By combining mutagenesis and electrophysiology with the use of azisevoflurane, a photoaffinity ligand of sevoflurane, Leu317 within the internal S4-S5 linker of the Kv1.2 channel has been identified as the sevoflurane binding site (Fig. 1D) [21, 22]. Azisevoflurane also labels a second distinct site, Thr384, near the external selectivity filter in the Kv1.2 G329T mutant, and this alters the voltage-dependent gating behavior [21]. These results indicate that the positive allosteric modulation of Kv channels by sevoflurane involves separate processes and multiple sites related to gating modification and the transition of channel state conformation. On the other hand, research has shown that the Kv1.2 channel is resistant to the intravenous anesthetic propofol. But AziPm, a photoactive propofol analog, inhibits the Kv1.2 channel while potentiating the Kv1.2 G329T mutant channel [23]. These results suggest that propofol likely binds to the Kv1.2 channel.

*In vivo* studies have shown that changing the activity of the Shaker-related K^+^ channel indeed alters the arousal state of unconscious animals induced by inhaled anesthetics. A microinfusion of an antibody against the Kv1.2 channel into the central medial thalamus (CMT) awakens 17% of rats anesthetized with 3.6% desflurane and 1.2% sevoflurane [24]. In addition, microinfusing a peptide inhibitor of Kv1.1, Kv1.3, and Kv1.6 channels into the CMT restores “consciousness” in rats anesthetized by 1.2% sevoflurane [20]. But the shaker K^+^ channel is not the target of xenon in short-sleeping *D. melanogaster* mutants [25].

Taken together, these data confirm that K^+^ channels in the Kv1.x family are important targets of volatile anesthetics (Table 2) and changing the activity of Kv channels in the CMT plays an important role in altering the arousal state in general anesthesia.

### Kv2.1–Kv2.2 (Shab) and Kv3.x (Shaw) Family Channels are Inhibited by Most Inhaled Anesthetics

The Kv2.x family includes two genes that encode the alpha subunit of the voltage-gated K^+^ channels named Kv2.1 and Kv2.2. They are also called Shab (Shaker cognate B)-related channels because of their homologous channel in *Drosophila* (Fig. 1A). The effects of anesthetics on these channels have also been studied. Kulkarni *et al*. reported that ketamine and halothane inhibit the Kv2.1 channel in a dose-dependent manner [26]. Recently, Kv2.1 channel activity was also shown to be inhibited by propofol *via* a mechanism that decreases its expression level in cortical neurons [27] (Table 3). Similar to Shab-related K^+^ channels, the voltage-gated Kv3.2 channel, a homolog of the K-Shaw2 channel, is also inhibited by alkanols and halogenated inhaled anesthetics such as halothane, isoflurane, chloroform, and desflurane [28]. Interestingly, the Kv3.2 channel is activated by sevoflurane (Table 1) [29]. Furthermore, many of the intravenous anesthetic drugs, such as thiopental, methohexital, propofol, midazolam, and droperidol (but not barbiturates or ketamine), also inhibit Shaw-like voltage-dependent K^+^ currents (Table 3) [30]. However, there are no *in vivo* data to show whether these channels change the arousal state under general anesthesia. The binding sites and inhibitory mechanisms of volatile anesthetics on these Kv channels remain unclear.

### Modulation of Other Voltage-Dependent Potassium Channels by Volatile Anesthetics

Among other Kv channels, Kv7.1 channel activity is inhibited to 59% by 190 µmol/L sevoflurane in Kv7.1-expressing cells, but the mechanism is still unclear [31]. Elk1, Kv7.2/7.3, Kv3.4, and Kv4.2 channels are resistant to volatile general anesthetics (Fig. 1A, B, Table 1) [32]. In addition, human ether-a-go-go-related gene (HERG) channels are inhibited by halothane through a mechanism that slows down their activation and accelerates their deactivation and inactivation (Table 1) [33]. Thus far, data to address the roles these channels play in general anesthesia *in vivo* are lacking. This demonstrates that the effects of general anesthetics on these Kv channels need further work.

## Modulation of K2P Channels by Inhaled Anesthetics

**Fig. 2:**
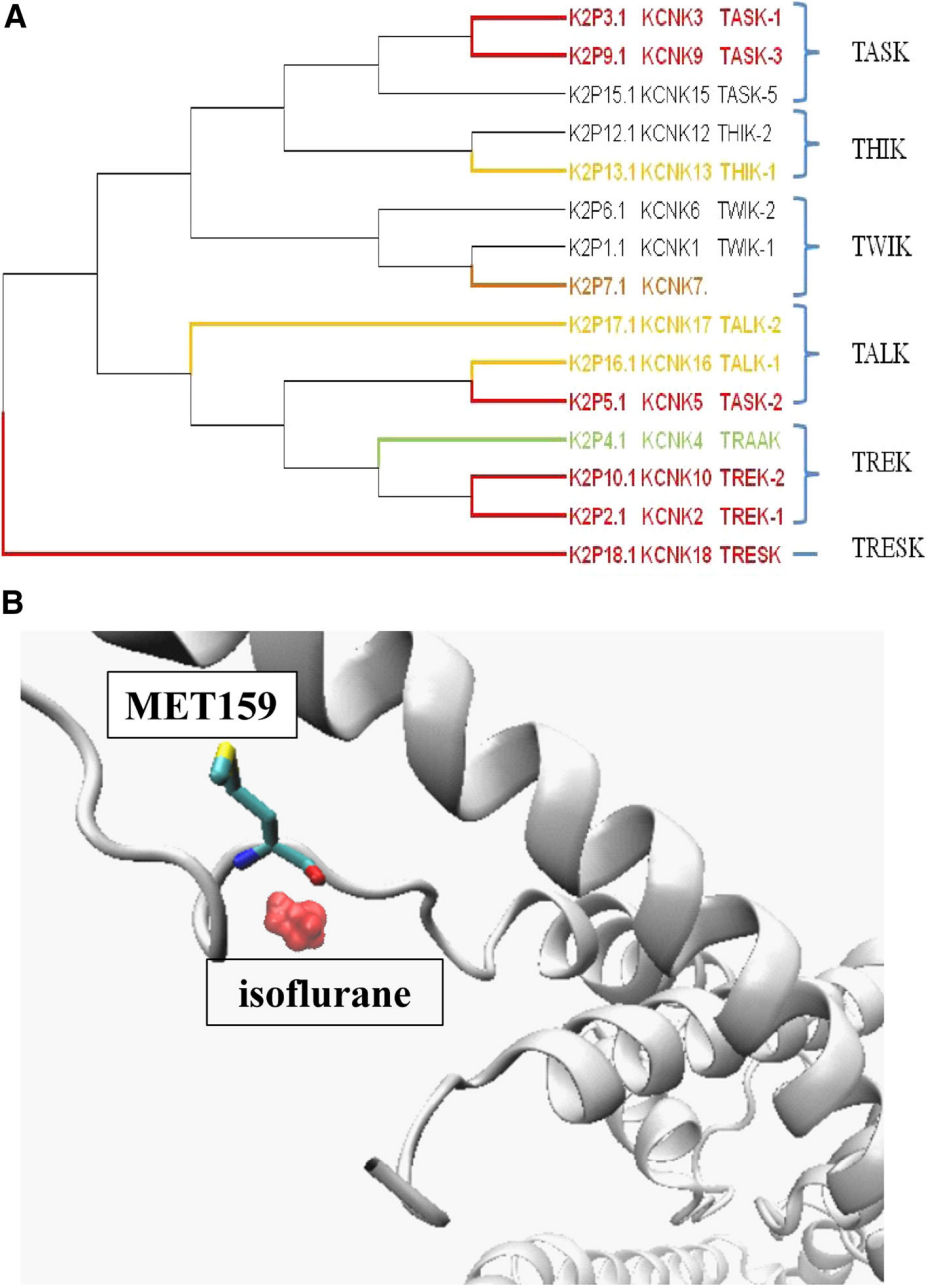
Phylogenetic tree of the K2P channel and local structure of TASK-1 channel for isoflurane binding. **A** TREK-1, TREK-2, TASK-1, TASK-2, TASK-3 and TRESK channels are activated by volatile anesthetics (red), whereas THIK-1, TALK-1, and TALK-2 channels are inhibited by volatile anesthetics *in vitro* (yellow). TRAAK channel activity (green) is not modulated by volatile general anesthetics. Responses of others channels (black) to anesthetics remain unknown. The protein IDs used in this alignment are as follows: K2P1.1: NP_002236, K2P2.1: NP_001017425, K2P3.1: NP_002237, K2P4.1: NP_201567, K2P5.1: NP_003731, K2P6.1: NP_004814, K2P7.1: NP_203133, K2P9.1: NP_001269463, K2P10.1: NP_066984, K2P12.1: NP_071338, K2P13.1: NP_071337, K2P15.1: NP_071753, K2P16.1: NP_115491, K2P17.1: NP_113648, and K2P18.1: NP_862823. **B** Local structure of K2P channel for isoflurane binding. The image was drawn based on the TASK-1 channel structure model by using the Swissport modeling system. The crystal structure of human two-pore-domain potassium ion channel TREK2 (K2P10.1) in complex with a brominated fluoxetine derivative was used as the template (crystal structure PDB file 4xdl.1).

The two-pore-domain K^+^ (K2P) channel family is composed of fifteen KCNK genes, each of which comprises four transmembrane segments and two pore domains in tandem. Two pore-forming subunits form a functional channel as dimers. K2P channels conduct time- and voltage-independent background, or ‘leak’ currents to generate a negative membrane potential in excitable and non-excitable cells. K2P channels can be classified into six subgroups (TREK, TASK, TWIK, TALK, THIK, and TRESK) based on coding genes and biophysical properties (Fig.  2A). The nomenclature provided by HGNC and IUPHAR, and a popular naming scheme are all assigned sequentially in Fig. 2 [34]. Among them, the TASK and TREK-1 channels were the first to be identified and they are potentiated by general anesthetics. Chloroform, diethyl ether, halothane, and isoflurane potentiate the TREK-1 channel, whereas halothane and isoflurane activate the TASK channel (Table 1) [35]. The C-terminus of the TREK-1 and TASK channels is important for activation by general anesthetics [35]. Subsequent studies have revealed many members of this family to be sensitive to volatile anesthetics. Here, we give an introduction according to the subfamilies.

### Inhaled Anesthetics Activate TASK-1 and TASK-3 Channels

Both the TASK-1 and TASK-3 channels are activated by clinically relevant concentrations of inhaled anesthetics [36]. Further studies have identified the amino-acid M159 as the critical determinant of the effect of isoflurane on the hTASK-1 channel; it is located between the region of the end of TM2 (transmembrane domain 2) and the middle of TM3 (Fig. 2B) [37]. But the mechanism by which the activity and conformation of the channel change has not been addressed. As a result, TASK-1-KO mice are less sensitive to the anesthetic effects of halothane, isoflurane, and dexmedetomidine than wild-type mice. They require higher concentrations of general anesthetics to induce immobility as reflected by loss of the tail-withdrawal reflex. TASK-3-KO mice also exhibit reduced sensitivity to the inhalation anesthetic halothane, but not isoflurane [36, 38] (Table 2). Although the TASK-3 channel is sensitive to some volatile anesthetics, it is insensitive to clinically-relevant concentrations of the anesthetic gases nitrous oxide, xenon, and cyclopropane (Table 1) [39]. The TASK-1 and TASK-3 channels are also inhibited by etomidate at concentrations used in clinical work but are insensitive to propofol [40] (Table 3). Furthermore, *in vivo* data suggest that periods of loss of the righting reflex produced by the same concentrations of propofol and pentobarbital in TASK-1-KO mice are longer than such periods in wild-type mice [41] (Table 4).

**Table 2:** Sensitivity changes toward volatile general anesthetics in potassium channel-knockout mice

	Halothane	Isoflurane	Sevoflurane	Desflurane	Chloroform	Xenon
K_v_1.1/1.2/1.3/1.6	–	–	Resistant	Resistant	–	No change
TASK-1	Resistant	Resistant	–	–	–	–
TASK-3	Resistant	Resistant	–	–	–	–
TREK-1	Resistant	–	Resistant	Resistant	Resistant	–
TASK-2	No change	No change	–	No change	–	–
KCNK7	No change	No change	–	No change	–	–
TRESK	No change	Resistant	No change	No change	–	–
GIRK2	No change	No change	–	No change	–	–

– unknown

**Table 3:** The effects of intravenous anesthetics on potassium channels

	Propofol	Etomidate	Katemine	Thiopental	Methohexital	Midazolam	Droperidol	Barbiturate	Chloral Hydrate	Trichloroethanol	Phenobarbital
K_v_2.1	Inhibition	–	Inhibition	–	–	–	–	–	–	–	–
K_v_3.2	Inhibition		No response	Inhibition	Inhibition	Inhibition	Inhibition	No response	–	–	–
K_v_7.2/7.3	No response	No response	No response	–	–	–	–	–	–	–	No response
K_v_11.1	No response	No response	No response	–	–	–	–	–	–	–	No response
K_v_12.1	No response	No response	No response	–	–	–	–	–	–	–	No response
TASK-1/3	No response	Inhibition	–	–	–	–	–	–	–	–	–
TREK-1/2	–	–	–	–	–	–	–	–	Activation	Activation	No response
TRAAK	–	–	–	–	–	–	–	–	Activation	Activation	No response
TRESK	No response	No response	–	No response	–	–	–	–	–	–	–
GIRK1/2 and GIRK2	No response	No response	No response	–	–	–	–	–	–	–	No response
GIRK1/4	No response	No response	No response	–	–	–	–	–	–	–	No response
Kir1.1and Kir2.1	No response	No response	No response	–	–	–	–	–	–	–	No response
BK	Activation	–	Inhibition	–	–	–	–	–	–	–	–
SK	–	–	Inhibition	–	Inhibition	–	–	Inhibition	–	–	–

– unknown

**Table 4:** Sensitivity changes toward intravenous general anesthetics in potassium channel-knockout mice

	Propofol	Phentobarbital	Dexmedetomidine	Diazepam
TASK-1	Sensitive	Sensitive	Resistant	Sensitive
TASK-3	No change	Unknown	No change	Unknown
TREK-1	Unknown	No change	Unknown	Unknown

### Activation of TREK Family Channels (TREK-1, TREK-2, and TRAAK) by General Anesthetics

Among the anesthetic-activated neuronal background K^+^ channels, the TASK and TREK channel subgroups have received the most attention [42]. The TREK channel is highly expressed in the human central nervous system, especially in the subventricular and ventricular zones, hippocampus, striatum, and certain parts of the cortex (layer IV) [43, 44]. The gating mechanisms of TREK channels include stretch, fatty acids, pH, and G protein-coupled receptors [45].

TREK-1 and its functional homologue TREK-2 are sensitive to volatile anesthetics such as chloroform, diethyl ether, halothane, and isoflurane (2–3 fold increase in current amplitude at 1 mmol/L anesthetic) (Table 1) [35]. However, the structurally and functionally related K2P channel TRAAK, which can even form functional heterodimeric channels with TREK-1, is insensitive to volatile anesthetics [35, 46]. This difference may be attributed to the specific structural features of transmembrane helix straightening and buckling of the M4 segment of the TRAAK channel (KCNK4) [47]. Another type of gaseous general anesthetic containing nitrous oxide and xenon has also been reported to open TREK-1 channels [39]. In addition, some intravenous anesthetics (chloral hydrate and trichloroethanol, but not phenobarbital) at pharmacologically relevant concentrations have been shown to open TREK-1 and TRAAK channels [48] (Table 3). Further, several *in vivo* experiments have demonstrated that the concentrations of some volatile anesthetics, including chloroform, halothane, sevoflurane, and desflurane, required for loss of the righting reflex and the tail clamp withdrawal reflex are significantly increased in TREK-1-KO mice [48, 49] (Table 2). Taken together, all these data suggest that TREK channels are important targets of general anesthetics.

### Modulation of the TALK Subfamily (TALK-1, TALK-2, and TASK-2), TWIK Subfamily (TWIK-1, TWIK-2, and KCNK7), THIK Family (THIK-1 and THIK-2), and TRESK Family (TRESK-1 and TRESK-2) Channels by General Anesthetics

The TASK-2 channel has low amino-acid identity with the TASK-1 and TASK-3 channels. The amino-acid identity and high sensitivity to pH suggest that the TASK-2 channel belongs to the TALK subfamily (Fig. 2A) [34]. In heterologous expression systems, a handful of experiments have been done to elucidate the pharmacological effects of volatile anesthetics on the TASK-2 channel. The results demonstrated that volatile anesthetics, among which chloroform was found to be the most potent, activate the TASK-2 channel, but not as potently as the TASK and TREK channels [50]. Thus, it is not surprising that the behavioral effects of inhaled anesthetics are unperturbed in TASK-2-KO mice [51] (Table 2). The minimum alveolar concentration (MAC) values of desflurane, halothane, and isoflurane in TASK-2-KO mice are similar to those of the wild-type [51]. Although *in vitro* studies have shown that TALK-1 and TALK-2 channel currents are inhibited by chloroform, halothane, and isoflurane, the possibility that they play a role in changing the arousal state in general anesthesia is low because they are mainly expressed in the pancreas [52].

The mRNA expression of TWIK-1 and TWIK-2 channels has been found in many brain regions [53], but their sensitivity to general anesthetics remains unknown (Fig. 2A). KCNK7-KO mice have been used to test the anesthetic effects of varying concentrations of isoflurane, sevoflurane, and desflurane, and the results showed that the MAC values of KCNK7-KO mice defined by the tail clamp withdrawal reflex are indistinguishable from those of the wild-type [54] (Table 2). Thus far, there is no evidence that this channel family is involved in changing the arousal state under general anesthesia.

THIK subfamily channels, including THIK-1 and THIK-2, were first cloned from rat brain and the amino-acid sequences of these two channels are 58% identical [55] (Fig. 2A). THIK-2 is expressed in most brain regions while THIK-1 is expressed in more restricted regions. However, the THIK-2 channel is less functional *in vivo* probably because its trafficking to the membrane is limited by putative retention/retrieval signals on both the N and C termini [55, 56]. The THIK-1 channel current is inhibited by inhaled anesthetics such as halothane [55]. In addition, *in vivo* data indicate that isoflurane causes activation of retrotrapezoid nucleus neurons *via* inhibition of a THIK-1-like background current [57], which suggests that the THIK-1 channel contributes to the maintenance of respiratory motor activity under general anesthesia.

The TRESK channel subfamily contains two members: TRESK-1 and TRESK-2 (Fig. 2A). Protein expression of the TRESK channel has been detected in many regions of the nervous system, including the dorsal root and trigeminal ganglia, cerebrum, cerebellum, brainstem, and sympathetic and parasympathetic ganglia, suggesting potential roles in pain transmission and anesthesia [58].

In the *Xenopus* oocyte and COS-7 cell expression systems, clinical doses of isoflurane, halothane, sevoflurane, and desflurane strongly increase TRESK currents up to three-fold by increasing the open probability [59]. Isoflurane is the most potent activator with an EC_50_ of ~ 150 µmol/L. However, some intravenous anesthetics, including etomidate, thiopental, and propofol, have little effect on TRESK channels [60] (Table 3). TRESK-KO mice have also been used to investigate the role of TRESK channels in volatile general anesthesia. The results showed that the MAC value of isoflurane is marginally increased (8%) while those of other volatile anesthetics are not significantly altered [61] (Table 2). These data suggest that either the role of the TRESK channel in general anesthesia is compensated by other channels in KO mice or the TRESK channel plays little role in altering the arousal state in general anesthesia.

## Modulation of Inwardly-Rectifying Potassium Channels by General Anesthetics

**Fig. 3:**
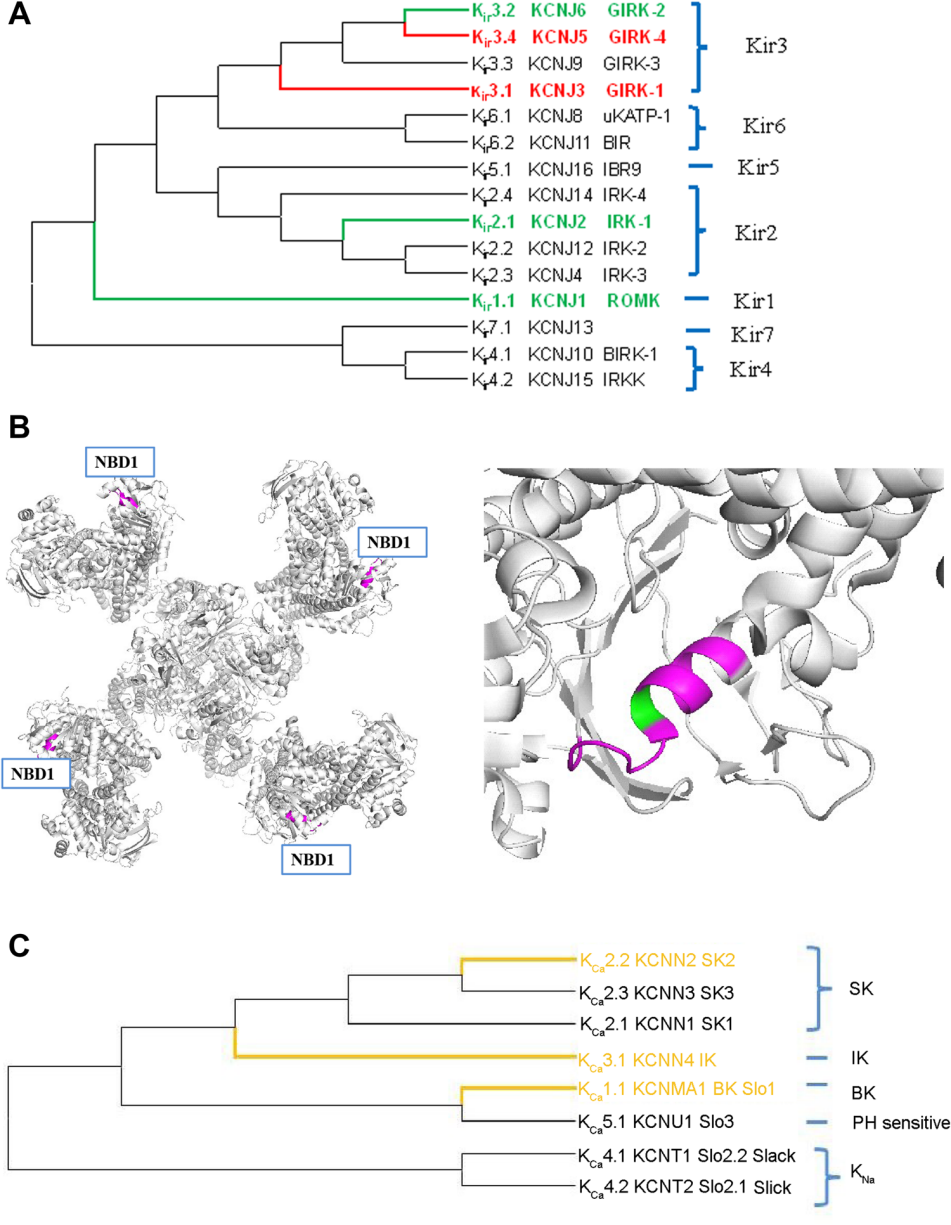
Phylogenetic trees of the Kir channels and the Ca^2+^-activated potassium channels and structure of NBD1 domain of Kir6.2 channel that alters the effect of isoflurane activation on the channel. **A** In Kir channel family, Girk1/Girk4 channels are activated by halothane (red). IRK1, Girk2 and ROMK channels are resistant to volatile anesthetics (green). Protein IDs of the Kir channel family are as follows: Kir1.1: NP_000211, Kir2.1: NP_000882, Kir3.1: NP_002230, Kir2.3: NP_004972, Kir3.4: NP_000881, Kir3.2: NP_002231, Kir6.1: NP_004973, Kir3.3: NP_004974, Kir4.1: NP_002232, Kir6.2: NP_000516, Kir2.2: NP_066292, Kir7.1: NP_002233, Kir2.4: NP_037480, Kir4.2: NP_733933, and Kir5.1: NP_001278554. **B** Left: Crystal structure of the Kir6.2 channel (based on PDB file 5TWV) with regulatory sulfonylurea receptor (SUR). The NBD1 domain (nucleotide binding domain) located on the SUR receptor is shown in purple color. Right: local structure of NBD1 domain with K708 residue that alters the affinity to isoflurane. **C** The phylogenetic tree of the Ca^2+^-activated potassium channels includes large-conductance channels K_Ca_1.1 (BK or Slo1), K_Ca_4.1 (Slack or Slo2.2), K_Ca_4.2 (Slick or Slo2.1), and K_Ca_5.1 (Slo3), intermediate-conductance channel K_Ca_3.1 (IK), and three small-conductance K_Ca_ channels are K_Ca_2.1–2.3 (SK). Of particular note, K_Ca_4.1–4.2 are activated by internal Na^+^ and Cl^−^; K_Ca_5.1 is activated by internal alkalization (OH). Channels that can be inhibited by volatile anesthetics are shown in yellow. Protein IDs are as follows: K_Ca_1.1: NP_001309764, K_Ca_2.1: NP_002239, K_Ca_2.2: NP_067627, K_Ca_2.3: NP_002240, K_Ca_3.1: NP_002241, K_Ca_4.1: NP_065873, K_Ca_4.2: NP_940905, and K_Ca_5.1: NP_001027006.

The mammalian Kir channel family includes 16 genes that encode an α subunit which has two transmembrane segments (M1 and M2) with a pore loop in between (Fig. 3A). Kir channels conduct K^+^ currents more readily in the inward direction than in the outward direction, and they are activated by ligand-stimulated G protein-coupled receptors. With activation of these receptors, G-protein βγ subunits are released from heterotrimeric receptors to interact with GIRK (G protein-coupled inwardly-rectifying K^+^) channels and open them. Among these genes, Kir3.x genes (GIRK1–4), such as GIRK1, GIRK2, and GIRK3 are predominantly expressed in neurons while GIRK4 is mainly expressed in the heart [10]. In neurons, GIRK channels either form GIRK2 homotetramers or form heterotetramers of GIRK1, GIRK2, and/or GIRK3 channels [62]. Tetramer channels with different subunits demonstrate different sensitivity to volatile anesthetics. Halothane enhances background currents through hetero-oligomeric GIRK1/GIRK4 channels but not through homo-oligomeric GIRK2 channels. This activation of basal current also requires the presence of G-protein βγ subunits. In contrast to basal GIRK currents, the agonist-induced GIRK currents (*via* co-expressed M2 muscarinic receptors) are inhibited by halothane (Table 1). In GIRK1/GIRK4 channels, this inhibition is most pronounced at low anesthetic concentrations (0.1 mmol/L–0.3 mmol/L) and occurs also when channels are activated by guanosine-5’-O-(3-thio) triphosphate. However, high concentrations of halothane (0.9 mmol/L) enhance the agonist-induced currents [63] (Table 1). This increase in agonist-induced currents is never seen with GIRK2 homo-oligomeric channels. Subsequent studies using GIRK1 channel (Del 363) and GIRK2 channel (Del 356) mutants that lack the C-terminal ends confirmed that these different modulatory effects produced by halothane are determined by the C terminus [64]. GIRK channels are resistant to intravenous anesthetics and nitrous oxide gas anesthetic (Table 3) [32]. On the other hand, efforts to investigate the roles of GIRK channels in general anesthesia *in vivo* using GIRK2-KO mice indicated that the MACs for 50% immobility in the tail clamp test for adult wild-type and GIRK2-KO mice are not significantly different [51] (Table 2). This result suggests that there is insufficient evidence to support the hypothesis that GIRK channels are involved in altering the arousal state in general anesthesia.

Among the Kir channels, Kir6.2 has also been named a KATP channel because it is inhibited by intracellular ATP and is sensitive to intracellular pH. It is composed of the pore-forming inward rectifier Kir6.2 and the regulatory sulfonylurea receptor SUR2A or SUR1. Although isoflurane does not directly interact with the Kir6.2 channel, it interacts with the SUR2A receptor to activate the Kir6.2 channel at low pH (pH 6.8). However, it has no activation effect at pH 7.4. Also, isoflurane cannot activate Kir6.2 channels co-expressed with SUR1 receptors (Table 1). Site-directed mutagenesis in the Walker motif of the SUR2A receptor abolishes the activation of Kir6.2 channels by isoflurane [65, 66]. Based on the recently dissected Kir6.2 channel structure, the nucleotide-binding domain on the SUR receptor is shown in Fig. 3B [67]. This study indicated that isoflurane preferentially enhances opening of the cardiac KATP channel (Kir6.2/SUR2A), suggesting a role of the KATP channel in cardiac protection in the anesthetic state induced by halogenated anesthetics.

## Modulation of Calcium-Activated Potassium Channels by General Anesthetics

The K_Ca_ family can be divided into three categories: BK (big conductance), IK (intermediate conductance), and SK (small conductance) channels [68]. BK channels are all tetrameric; they are formed by α subunits that have 6/7 transmembrane segments encoded by genes *SLO1-3* [69]. But the Slo1 and Slo2 channels are expressed in brain, while the Slo3 channel is specifically expressed in testis and spermatozoa [70]. The Slo2 channel subfamily includes Slo2.1 and Slo2.2, which are activated by Na^+^ rather than Ca^2+^ [71]. The Slo1 channel is activated by both voltage and intracellular Ca^2+^ while the IK and SK channels are gated by intracellular Ca^2+^ alone. BK channels are diffusely expressed in almost the whole brain, whereas IK channels are mainly expressed in T lymphocytes, red blood cells, and parotid glands [72].

The regulatory effects of general anesthetics on BK, IK, and SK channels have been investigated. The volatile anesthetic halothane, at a clinically relevant dose of 0.5 mmol/L, reduces the open probability of BK channels without altering the single-channel conductance. However, this effect is blocked by increasing the concentration of cytoplasmic free Ca^2+^ from 1 µmol/L to 100 µmol/L, suggesting that halothane serves as a closed channel blocker [73]. In addition to halothane, isoflurane and enflurane also inhibit BK channels at a clinically relevant dose (Table 1) [74, 75]. Also, the intravenous anesthetic ketamine (at clinically relevant concentrations: 2–500 µmol/L) selectively blocks the BK channel in a dose-dependent manner. Ketamine shifts the open probability *vs* voltage curve to a higher potential without changing the slope of the voltage-dependence [76]. Interestingly, another intravenous anesthetic (propofol) activates the BK channel to relax coronary arteries [77]. A study using animal models showed that Slo-1-mutated *Caenorhabditis elegans* are resistant to volatile anesthetics [78]. But the effect of general anesthetics on Slo1-KO mice has not been reported.

IK channel currents are rapidly and reversibly inhibited by many volatile anesthetics such as halothane, isoflurane, enflurane, and sevoflurane with an EC_50_ from 0.4 to 1 mmol/L, whereas the SK channel is not inhibited by volatile anesthetics [79, 80]. However, the SK channel is blocked by the intravenous anesthetics ketamine, barbital, and methohexital [80]. Experiments using patch clamp in brain slices have further confirmed that propofol enhances the inhibition of spike firing in retrotrapezoid nucleus neurons by blocking SK channels (Table 3) [81].

Although the effects of general anesthetics on Ca^2+^-activated K^+^ channels have been investigated, the underlying mechanism and the overall roles played by these channels in general anesthesia remain to be explored.

## Concluding Remarks and Future Perspectives

In summary, K^+^ channel activity is widely regulated by general anesthetics. Among them, the mechanisms by which Kv1.2, Kir6.2, and TASK-1 channels are modulated by general anesthetics have been studied in detail. Local structures and amino-acids critical for anesthetic binding to Kv1.2, Kir6.2, and TASK-1 channels revealed by biophysical studies have further clarified the characteristics of the molecular interactions of general anesthetics with these K^+^ channels. In addition, recent research has confirmed that activity changes in Kv1.x, TASK-1, TASK-3, and TREK-1 channels are essential for altering the arousal state when volatile anesthetics play their roles (Tables 2 and 4). In addition, the role of the THIK-1 channel in the retrotrapezoid nucleus is to maintain respiratory motor activity under general anesthesia. However, the biophysical mechanisms of the regulatory effects of general anesthetics on other K^+^ channels remain to be further explored. In the meantime, the roles of many K^+^ channels in general anesthesia remain elusive, especially the nuclei and neuronal circuits in which they play their roles. *In vitro* biophysical studies combined with optogenetic electrophysiological studies *in vivo* using K^+^ channel KO mice will greatly facilitate further addressing the role of K^+^ channels in general anesthesia.
